# Mercury in Syphilis

**Published:** 1874-04

**Authors:** 


### Mercury in Syphilis.

The following are Mr. Jonathan Hutchinson's *conclusiors*, as given in the London Lancet:

That mercury is probably a true vital antidote against the syphilitic virus, and that it is capable of bringing about a real cure.

That in practice a good many cases are really cured by mercury; the cure being proved by the restoration to good health, and in some cases by renewed susceptibility to contagion.

That the probability of cure depends upon the stage of development attained by the disease when the remedy is resorted to, and upon the perseverance with which it is used.

That in order to secure the antidotal efficacy of mercury against syphilis, it is desirable to introduce a considerable quantity into the system and to protract its use over a very long time.

That ptyalism and other evidences of the physiological action, so for from being beneficial, are, if possible, to be carefully avoided, since they prevent the sufficiently prolonged use of the remedy.

That in cases in which the patient shows an idiosyncrasy peculiarly susceptible to mercury, the indication is to reduce the dose rather than to omit the drug.

That it is impossible to begin the administration of mercury too soon, and that it should be resorted to without loss of time in all cases in which a chancre shows a tendency to indurate.

That many cases of indurated chancre treated early by mercury never show any of the characteristic symptoms of the secondary stage.

That in other cases of mercurial cure of the chancres in which yet secondary symptoms do occur, they are usually milder than if allowed to develop without specific treatment.

That when mercury does not wholly abrogate the secondary stage it exhibits a remarkable power in delaying it.

That delayed outbreaks of secondary syphilis are to be regarded rather as a proof that the administration has not been sufficiently persevering than that the remedy was not efficient.

That it is probable that the rise of tertiary symptoms is in ratio with the severity and prolonged duration of the secondary stage.

That there are some grounds for believing that the tertiary symptoms of syphilis are both less frequent and less severe in those who have been efficiently treated by mercury than in others.

That mercury cautiously given does not in a great majority of instances do any injury to the general health, and that its local inconveniences may usually be prevented.

That the doctrine of the real antidotal character of mercury in respect to syphilis ought to lead to much more prolonged administration of it, with the hope of destroying utterly all lingering germs of the malady.

That most collected statistics as to the duration of treatment and freedom from relapse are misleading and worse than useless, because usually the treatment was far too short to be effectual.

That it has not yet been proved that there are any special forms of syphilitic disea es in which mercury ought to be avoided, although, as a general rule, it is acknowledged that it must be used with more caution in all forms which are attended by ulceration than in others.

That iodide of potassium possesses little or no efficacy against either the primary or secondary forms of syphilis.

That the efficacy of mercury is often most signally proved in cases which have utterly resisted the action of iodide of potassium.

That it does not matter whether mercury is given by the mouth, by inunction, or by the vapor-bath, provided that whichever method be selected care be taken to avoid salivation, purging, etc.

That the doses usually resorted to for internal administration are for the most part too large, and thus often necessitate a premature discontinuance of the remedy.

That if one method of administration does not proceed satisfactorily, another should be tried, and that in no case of difficulty should the vapor-bath be forgotten.—"*The Practitioner.*"

